# Lipid-Based Nanocarriers: Bridging Diagnosis and Cancer Therapy

**DOI:** 10.3390/pharmaceutics16091158

**Published:** 2024-09-01

**Authors:** Alessandra Giordano, Anna Chiara Provenza, Giorgio Reverchon, Lucia Baldino, Ernesto Reverchon

**Affiliations:** 1 Department of Industrial Engineering, University of Salerno, Via Giovanni Paolo II, 132, 84084 Fisciano, Italy; a.giordano219@studenti.unisa.it (A.G.); a.provenza2@studenti.unisa.it (A.C.P.); ereverchon@unisa.it (E.R.); 2 Diagnostic and Interventional Radiology, IRCCS Istituto Ortopedico Rizzoli, Via G.C. Pupilli, 1, 40136 Bologna, Italy; giorgio.reverchon@gmail.com

**Keywords:** theranostics, lipid-based nanocarriers, lipid nanocapsules, micelles, solid-lipid nanoparticles, nanoemulsions

## Abstract

Theranostics is a growing field that matches diagnostics and therapeutics. In this approach, drugs and techniques are uniquely coupled to diagnose and treat medical conditions synergically or sequentially. By integrating diagnostic and treatment functions in a single platform, the aim of theranostics is to improve precision medicine by tailoring treatments based on real-time information. In this context, lipid-based nanocarriers have attracted great scientific attention due to their biodegradability, biocompatibility, and targeting capabilities. The present review highlights the latest research advances in the field of lipid-based nanocarriers for cancer theranostics, exploring several ways of improving in vivo performance and addressing associated challenges. These nanocarriers have significant potential to create new perspectives in the field of nanomedicine and offer promise for a significant step towards more personalized and precise medicine, reducing side effects and improving clinical outcomes for patients. This review also presents the actual barriers to and the possible challenges in the use of nanoparticles in the theranostic field, such as regulatory hurdles, high costs, and technological integration. Addressing these issues through a multidisciplinary and collaborative approach among institutions could be essential for advancing lipid nanocarriers in the theranostic field. Such collaborations can leverage diverse expertise and resources, fostering innovation and overcoming the complex challenges associated with clinical translation. This approach will be crucial for realizing the full potential of lipid-based nanocarriers in precision medicine.

## 1. Introduction

Cancer is a significant health challenge at global level [1]. Each year, 19–20 million people worldwide are diagnosed with cancer and approximately 10 million deaths are related to this disease, placing it as the second cause of death in the world [2]. According to estimates from the World Health Organization (WHO), cancer-related deaths could exceed 11 million by the year 2030. There are over 200 distinct types of cancer, each classified according to specific cell types [3]. Therefore, the need for advanced technologies, which would facilitate the early detection of these pathological states, is a crucial aspect to perform timely treatment.

Over the past decades, the scientific community engaged in cancer research has emphasized multidisciplinary approaches for cancer treatment, with the aim of achieving maximum efficiency in the cure [3]. In this context, nanotechnology plays a central role, notably in the development of nanoparticles intended for both imaging and therapy, and this field is known as theranostics [4].

Theranostics is a growing field that uses imaging techniques to monitor the real-time effectiveness and safety of therapies targeting complex diseases, such as cancer [5]. Within nanotheranostics, there are diagnostic and therapeutic applications. For diagnostic applications, non-invasive techniques can be investigated, such as computed tomography (CT), magnetic resonance imaging (MRI), near-infrared imaging (NIR), ultrasound imaging (USI), and positron imaging tomography (PET). In the fight against cancer, therapeutic applications involving nanotheranostic agents can be categorized into three main approaches, including photodynamic therapy (PDT), photothermal therapy (PTT), and chemotherapy [6]. Additionally, other emerging approaches include magneto-thermal therapy, ultrasound-responsive therapy, and radiotherapy [7].

In Table 1, a brief overview of the imaging and treatment modalities mentioned above is reported.

With the goal of promoting more targeted and personalized disease management [40], the aim in theranostics is to administer the treatment in the precise moment and in the exact dosage, optimizing effectiveness while minimizing risks [41]. Moreover, imaging agents can be significantly helpful in predicting the destination or in vivo pathway of a drug in systemic circulation and can also be useful in determining the pharmacokinetics of an anticancer formulation [42]. In this scenario, nanoparticles serve as a versatile platform that can integrate distinct substances simultaneously, such as therapeutic agents, i.e., chemotherapeutic agents, gene therapy, photothermal therapy, and photodynamic therapy, using various types of imaging agent, such as fluorescent dyes, fluorescent polymers, quantum dots, metal nanoparticles, and magnetic nanoparticles, in a single platform [43]. Among the various nanoformulations adopted in the oncology field, lipid-based nanocarriers have received considerable attention in view of their biodegradability, biocompatibility, and targeting capabilities [44].

Lipid-based nanoparticles have significant clinical relevance and potential impact in the field of theranostics. These particles combine both diagnostic and therapeutic functions, allowing for the precise targeting of cancer cells while minimizing harm to healthy tissues. Their biocompatibility and ability to encapsulate a wide range of therapeutic agents make them ideal for personalized medicine. Lipid-based nanoparticles have the potential to enhance the delivery and efficacy of chemotherapeutic drugs, mitigate side effects, and enhance imaging capabilities, thereby providing a comprehensive approach to cancer treatment. The incorporation of these nanoparticles into clinical practice has the potential to revolutionize cancer therapy by providing more efficient and less invasive treatment alternatives for patients.

Therefore, this review presents an outline of the different lipidic nanoparticles developed for cancer imaging and treatment, including liposomes, solid-lipid nanoparticles (SLNs), nanostructured lipid carriers (NLCs), lipid nanocapsules, lipid nanoemulsions (NE), and lipid micelles. The focus is on design considerations for creating therapeutic particles, and the main advantages of these lipid-based theranostic agents over other types of particles used for imaging and targeted cancer therapy. This work is intended to fill the gap in the literature with a detailed description of different types of lipid nanoparticles specifically utilized for theranostics and their trial status in this emerging strategy. Therefore, the main goal of this review is to highlight the current challenges in the development of lipid-based nanocarriers and possible approaches to overcoming them in order to achieve the translation to the clinical phase of these advanced nanomaterials.

## 2. Key Parameters in Lipid Nanoparticles Design for Theranostics

Over the past few years, various types of nanoparticles, including magnetic [45,46,47], metallic [48,49,50], polymeric [51,52], quantum dots [53,54], carbon nanomaterials [55,56], dendrimers [57], and lipidic nanoparticles [58,59], have all been successfully designed for the treatment of cancer. Their distinct chemical, mechanical, electrical, optical, magnetic, electro-optical, and magneto-optical characteristics [41] make them attractive for biomedical applications, especially in cancer diagnosis, treatment, and monitoring. However, despite numerous advancements, drug delivery systems (DDSs) still face safety challenges. As a result, safer DDSs of biocompatible substances, such as lipid carriers, are generally preferred.

Lipid-based nanoparticles, consisting of various lipid components, have been proposed as safer candidates for cancer therapy due to their similarity to biomembranes and biocompatibility [60]. During the formulation phase of these nanocarriers, in vivo stability is crucial, as they encounter harsh conditions within the tumor microenvironment after administration. The interaction of nanoparticles with biological materials poses challenges for the transfer of lipid-based theranostic nanomedicine to clinics, given their potential toxicity or incompatibility. Toxicity depends on parameters such as nanoformulation solubility, size, and zeta potential [61]. Indeed, upon entering a biological system, nanoparticles tend to interact with proteins, resulting in the formation of a “corona” on their surface. This minimizes the surface energy of the nanomaterial, and significantly alters its chemical composition, physicochemical interactions, and biological responses [62]. For instance, nanoparticles that bind opsonins with high affinity are quickly cleared from the bloodstream [61]. Therefore, it is essential to functionalize surface nanoparticles in order to extend their blood circulation time and enhance their bioavailability. Hence, it is understood that the effective delivery of drugs or diagnostic markers to target cells relies on optimizing the design and structure of these nanodevices. In this sense, nanoparticle size, polydispersity, charge, and surface modification play pivotal roles in designing theranostic systems.

### 2.1. Structure, Design, and Geometry of Nanoparticles

Nanoparticles’ geometry (i.e., shape and size) is a crucial factor in determining their functions, such as encapsulation capacity, drug release, stability, cytotoxicity, and delivery performance. Nanoparticles are characterized by a high surface-area-to-volume ratio, promoting the surface conjugation of functional parts [63]. Dimensions, morphology, surface modification, and surface charge are critical characteristics that influence the distribution, flow, long-term safety, and toxicity potential of nanomaterials [64]. In terms of design, nanosystems with diameters from 5 to 200 nm are suitable for tumor targeting; it is important to consider that this assumption depends on the kind of cancer and its characteristics. The dimension determines encapsulation capacity, stability, and interaction with the biosurface. Smaller diameters (80–200 nm) are related to increased stability, whereas larger nanoparticles exhibit higher encapsulation capacity, although they are less stable and more readily identified by the immune system. Therefore, the mean dimension of nanoparticles should present an equilibrium between an enhanced encapsulation capacity (increased diameter) and successful pharmacokinetics (smaller dimension) [65].

Historically, the most traditional drug delivery carriers were based on spherical systems. However, researchers are interested in non-spherical systems, such as extended or filamentous shapes. These new carriers range from elongated liposomes to stretched, flat disks or filamentous micelles. In particular, different geometries are related to different aspect ratios that determine different behaviors of nanoparticles (NPs) in the blood. For example, elongated structures, filomicelles, and flexible carriers, due to their aerodynamic properties and their shape, are more easily subjected to the action of the surrounding fluid force. This generates a collision with the vascular wall, increasing the interaction with macrophages and the rate of elimination by the immune system. On the other hand, non-spherical carriers could provide advantages for drug targeting because they have a large available surface area to conjugate targeting agents. Additionally, the smaller size of non-spherical nanocarriers allows for efficient perfusion through capillaries, improving access to multiple targets that are unreachable by micron-sized spherical particles [63]. The increased permeability and retention effect (EPR effect) is associated with a phenomenon and process whereby macromolecular components can subsequently gather in the vascularized cancer area, thus achieving the targeted delivery and retention of anticancer agents in solid tumor tissue [66]. The EPR effect constitutes the main base for passive targeting to increase drug-loaded NPs in cancers. Although many parameters can affect the EPR effect, particle diameter, form, and surface charge are the main nanoparticle factors that should be considered for increasing it [67]. The surface charge is a crucial physical and chemical characteristic that can influence cellular uptake, cell viability, and pharmacokinetics [68]. Specifically, it has been observed that nanoparticles exhibit a tendency to aggregate when their charge is near neutral, whereas those with a high positive charge are more readily absorbed by macrophages. Therefore, lipid-based nanoparticles with a negative charge might have superior performance [69].

### 2.2. Surface Chemistry

The surface chemistry can determine the mechanism of action between nanoparticles and cell membranes and the cytotoxicity. In fact, surface chemistry is the principal property moderating the cellular toxicity of nanoparticles, and it includes the surface charge and surface coating. Positively charged nanoparticles improve biocompatibility towards cells; the surface charge is measured by the zeta potential parameter. The composition of the surface chemistry of nanoparticles improves affinity for target therapy [70]. Indeed, the surfaces of nanoparticles can be functionalized by incorporating invisible or targeted parts, which can improve bioavailability and target capacity, decreasing nanoparticle cytotoxicity. Notably, to enhance the circulation properties of carriers in the bloodstream, they are coated with polyethylene glycol (PEG), which contributes to reduction interactions with the immune system [63]; other approaches are also proposed through different surface modifications, specific biological targeting, imaging, and diagnostic components, including antibodies, ligands, peptides, proteins, aptamers, small molecules, and carbohydrates [71,72]. These components are aimed at precise receptors on the surfaces of tumor cells, minimizing non-defined toxicological effects on normal cells [41]. Therefore, the surfaces of lipid-based nanocarriers can be decorated with a variety of targeting ligands, contingent upon the intended biomedical application. Nonetheless, it is interesting to observe that the functionalization procedure itself can significantly impact the effectiveness of targeting ligands. In the following section, the most widely adopted strategies for functionalization are explored.

### 2.3. Surface Functionalization Strategies

PEG-coated lipid-based nanocarriers provide special long-circulation properties, which are also desirable in the development of lipid-targeted nanocarrier formulations. The utilization of PEG on nanocarrier surfaces is a commonly employed technique in the process of coupling a particular ligand to the carrier. At least four primary methods for the functionalization of lipid-based nanocarriers have been proposed in the literature, as follows: (1) the direct attachment of the ligand to pre-existing nanocarriers, (2) incorporating a lipid–PEG–ligand complex during the nanocarrier formulation, (3) the post-insertion of lipid–PEG–ligand micelles, and (4) the noncovalent adsorption of the ligand onto the surfaces of lipid-based nanocarriers.

(1) Direct attachment of the ligand to pre-existing nanocarriers. The direct attachment of ligands to existing nanocarriers is a method in which the ligands are bound to the nanocarrier surface via lipid heads or PEG chains. This technique relies on a series of chemical reactions for covalent bonding, such as the formation of amide bonds, the formation of thioester bonds through the addition of maleimide–thiol, the construction of disulfide bridges, hydrazone linkage, and the utilization of biorthogonal chemistry. This strategy has a major advantage because it does not require the alteration of the nanocarriers’ composition before the conjugation process. However, it requires reactive groups on the nanoparticle surface to establish a covalent bond [73,74].
(2) Incorporating a lipid–PEG–ligand complex during the nanocarrier formulation. In this protocol, first, the lipid–PEG–ligand complex is synthesized. Following this, the conjugate is combined with structural lipids using a one-pot assembly technique in order to produce targeted lipid nanoparticles. This method facilitates the practical adjustment of ligand density by regulating the proportion of targeting ligands incorporated. However, it has been demonstrated that a significant fraction of targeting ligands end up facing the internal cavities of the lipid nanoparticles, making them inaccessible for active targeting purposes [73,74,75,76].
(3) Post-insertion of lipid–PEG–ligand micelles. This strategy for the preparation of targeted lipid nanoparticles is advantageous for its simplicity and the stability it confers to the nanoparticles. It is based on the amphiphilicity of the targeted ligands, whose hydrophobic chains can be incorporated into the lipid bilayers of preformed nanoparticles, while the hydrophilic heads remain exposed to the aqueous environment. The process, commonly referred to as the post-insertion method, involves the incubation of preformed nanoparticles with micelles formed by PEG–lipids, which are amphiphilic compounds that self-assemble into micelles above their critical micelle concentration (CMC). Under controlled conditions, PEG–lipid conjugates can be transferred into the lipid layer of the nanoparticles. After incorporating the PEG–lipid, a chemical reaction can be used to covalently attach the ligands to the nanoparticle surface, ensuring the stability and specificity of the targeting. However, the implementation of such a protocol may result in the existence of residual reactive end groups on the surface that may cross-link and, consequently, facilitate a more expeditious elimination from the blood circulation. Furthermore, unreacted groups located on the internal surface of the particles may undergo undesirable interactions with drug molecules or other lipid components. Hence, an alternative approach involves the pre-attachment of the ligand to the PEG–lipid prior to its incorporation into lipid-based nanoparticles. This approach can prove to be more advantageous as it guarantees the presence of the ligand on the nanoparticle surface. This method is particularly beneficial because it allows for the optimization of ligand insertion conditions separately from drug preparation and loading, leading to high conjugation efficiency and ensuring that ligands are positioned on the external surfaces of lipid-based nanoparticles [73,74,75,77].
(4) Noncovalent adsorption of the ligand onto the surfaces of lipid-based nanocarriers. This method uses techniques such as physical adsorption or ionic bonding to functionalize lipid-based nanocarriers. In physical adsorption, ligands attach to the surface through weak interaction forces, such as electrostatic interactions, hydrogen bonds, hydrophobic interactions, and van der Waals forces. The advantages of noncovalent adsorption encompass rapid functionalization, versatility in ligand types, and reversibility. However, the stability of noncovalent adsorption is a major concern, especially for in vivo applications where lipid-based nanocarriers must maintain functionality in complex and dynamic environments. Conjugation techniques, which generate stronger and more durable bonds between the ligand and the nanoparticle, are favored for long-term applications or when greater stability is required [73,75,78].

## 3. Case Studies: Lipid Nanoparticles in Theranostic Field

**Figure 1:**
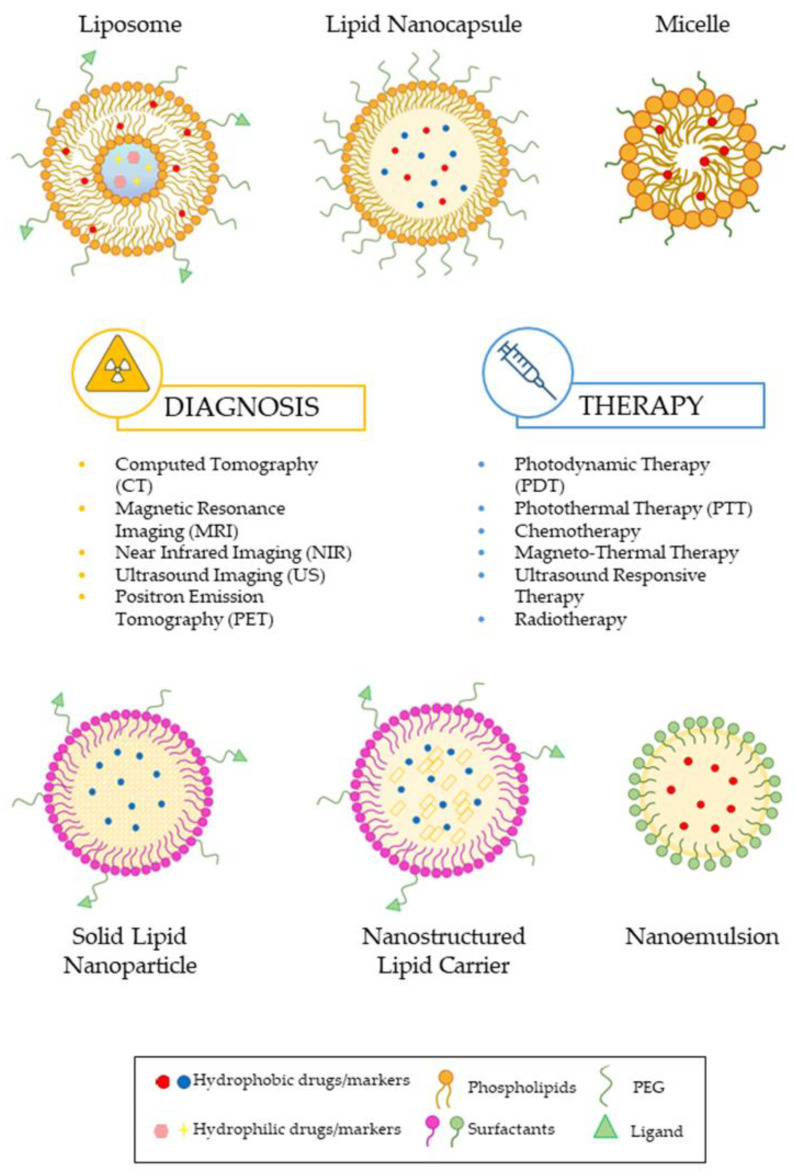
Schematic composition and organization of different types of LNPs described in this work.

Lipid nanoparticles (LNPs) are widely employed as carriers for the delivery of therapeutic agents. LNP-based delivery systems can overcome the limitations associated with other drug delivery carriers, thanks to their small diameter, biocompatibility, and biodegradability [79]. LNPs have been engaged to deliver chemotherapeutic drugs and to mitigate the toxicity in cancer patients. They represent a powerful system for drug administration in oncology, thanks to the presence of phospholipids in their structure, which provide them with unique physical and chemical characteristics [80]. In Figure 1, different types of LNPs for theranostic applications in both diagnosis and the therapeutic field are represented. The case studies investigated in this review were selected on Google Scholar and Scopus by choosing works from the last decade.

### 3.1. Liposomes

Liposomes were discovered in 1965 and are the reference model for the production of lipid vesicles to obtain an effective drug/diagnostic delivery, thanks to their dimensions, hydrophilic and hydrophobic properties, biodegradability, biocompatibility, low toxicity, and low immunogenicity [81]. Liposomes with a mean size of between 80 and 200 nm, neutrally or negatively charged, are mainly used for theranostics thanks to their high stability [3].

For imaging purposes, nanoscale diagnostic agents can be encapsulated inside theranostic liposomes, and the active principles can either be encapsulated in the core or fixed in the lipophilic shell. In order to achieve a specific targeting drug delivery, the surfaces of liposomes can be modified by adding an agent (i.e., PEG) on the external lipophilic bilayer, which increases the biocompatibility and hides them from the immune system [82]. The diameter and lipid configuration of liposomes are key parameters that influence their circulation time (half-life) in the system. Larger lipid nanovesicles exhibit a faster clearance rate compared to smaller ones, as their larger surface area allows for greater serum protein absorption (opsonization). Liposomes with a diameter larger than 200 nm are primarily eliminated by macrophages, while those with a size of less than 70 nm are principally cleared by cells [83].

Several studies are concentrated on the production and the optimization of liposomes for theranostic applications. For example, Lozano et al. [84] produced targeted and non-targeted PEGylated liposome-ICG (indocyanine green) to encapsulate the anticancer drug, doxorubicin (DOX), for therapeutic and diagnostic applications. The DOX was encapsulated in the hydration step along with the incorporation of the ICG. The mean diameter increased from 110 nm to 130 nm after post-insertion, and the PDI increased from 0.018 to 0.115. The zeta potential was negative and increased in absolute value after insertion from −25.8 ± 1.4 mV to −39.4 ± 1.0 mV.

ICG was also used in the study by Shemesh and colleagues [85] as a photosensitizer. Their goal was to produce and study a theranostic liposomal carrier using ICG, activated by NIR irradiation, for the in vivo PDT of breast cancer. The mean hydrodynamic diameter of the liposomal indocyanine green (LPICG) was about 80 ± 12 nm. In summary, the researchers proved that LPICG could improve the anti-tumor therapeutic efficacy of PDT in tumor-bearing mice, resulting in high tolerability and cancer elimination.

Thébault et al. [86] proposed an innovative therapeutic approach involving the magnetic accumulation of Ultra Magnetic Liposomes (UML) followed by High-Intensity Focused Ultrasound (HIFU). This strategy was designed to study an antivascular agent, controlled by MRI. To achieve this goal, the authors co-encapsulated combretastatin A4 phosphate (CA4P), an antivascular agent, within the UML, resulting in CA4P-loaded thermosensitive Ultra Magnetic Liposomes (CA4P-UMLs). The liposome sizes were 230 ± 46 nm for the UMLs and 209 ± 56 nm for the CA4P-UMLs. The reduction in the cancer volume was achieved thanks to the complete combination of theranostic liposome encapsulating CA4P, magnetic targeting, and precise ultrasound release. MRI bioimaging evidenced the efficiency of this therapeutic application in vivo, with the functional alteration of the vascularization by the decreased permeability, as well as reductions in tumor growth.

Liposomes containing gadolinium (Gd) complexes (Gd-lips) co-encapsulated with an active principle can help with either imaging or treatment in different pathologies. Šimečková et al. [87] produced Gd-lip containing PE-DTPA chelating Gd^+3^ by using the thin-film hydration method. The Gd–liposomes (Gd-lips) showed no cytotoxicity to human liver cells and were characterized by an average size equal to 113 ± 0.5 nm, a PDI equal to 0.04, and a zeta potential of −57.6 ± 1.9 mV.

Karpuz et al. [88] formulated liposomes encapsulating paclitaxel (PTX) and vinorelbine (VNB), and examined the in vivo therapeutic and diagnostic effects of lipid nanosystems through the evaluation of the biodistribution, tumor growth rate, and in vivo histopathologic examination of lung carcinoma cells. The lipid nanovesicles showed wide cellular uptake; however, the co-drug-encapsulating liposomes showed a larger cytotoxicity profile than the free drug combination in the lung carcinoma cells. The particle sizes of the folate-targeted liposomes (between 140 and 190 nm) were larger than those of the untargeted liposomes (from 120 to 150 nm). The PDI values were between 0.11 and 0.21; the zeta potential values ranged between −7.9 mV and −9.3 mV. The encapsulation efficiencies of the lipid nanovesicles were 15% for the PTX and 20% for the VNB.

In another work, Karpuz and coworkers [89] prepared neutral and cationic liposomes loaded with imatinib nesylate (IMT) and labeled with ^68^Gallium (Ga) for the PET imaging and treatment of breast cancer. The liposomes exhibited mean diameters between 133 nm and 250 nm, with a PDI lower than 0.4. The zeta potential values of the lipid formulations were placed in a wide range (from −13 to +54 mV). Therefore, based on the findings of this study, ^68^Ga-Lipo/IMT was a suitable theranostic agent for breast cancer imaging and therapy, owing to its excellent properties, low cytotoxic profile, and strong cellular binding ability.

The critical examination of these works highlights that liposomes are widely used kinds of carriers for theranostic applications, providing advantages in targeted drug delivery, controlled release, and enhanced imaging capabilities. However, in the future, they will face several challenges related to stability, production methods, and rapid clearance from the bloodstream, in order to achieve their full potential in the theranostic field.

### 3.2. Solid-Lipid Nanoparticles (SLNs)

Solid-lipid nanoparticles (SLNs), introduced in 1991, have emerged as efficient drug carriers and as alternatives to other delivery systems, such as emulsions, liposomes, and polymeric nanoparticles [90]. SLNs are colloidal carriers with nanoscopic sizes ranging from 50 to 1000 nm. They are typically spherical in shape and consist of a solid-lipid core (maintaining stability at both room and body temperatures), surrounded by a layer of interfacial surfactant or polymers [91,92]. Generally, the SLN matrix can be composed of a broad range of physiological lipids with low toxic potential, including fatty acids (e.g., stearic acid, palmitic acid), triglycerides (e.g., trilaurin, tripalmitin, and tristearin), and saturated fatty acids (e.g., glycerol behenate and cetylpalmitate) [93]. SLN production is mainly based on solidified nanoemulsion technologies, including high-pressure homogenization, high shear homogenization, ultrasonication, solvent emulsification–evaporation, and “coacervation” [94]. They are generally prepared by heating a lipid mixture above its melting point, adding the drug, mixing, and finally cooling to immobilize the drug within the solid-lipid spheres [93]. SLNs show several attractive features, including low toxicity, large surface area, controlled drug release, and high cellular uptake [58,95]. In addition, they can encapsulate lipophilic, hydrophilic, amphiphilic, and charged molecules [96,97]; therefore, they can efficiently deliver contrast agents, as well as anticancer drugs, allowing simultaneous treatment and diagnosis, as evidenced by the results of recent research studies.

Bae and coworkers [24] proposed the use of solid-lipid nanocarriers loaded with quantum dots (QDs) for anticancer theranostics, in combination with a mixture of PTX and siRNA. In this formulation, QDs and PTX were incorporated within the lipid matrix of SLNs, whereas anionic siRNA molecules were electrostatically conjugated to the outer cationic surfaces of nanoparticles. These paclitaxel–siRNA-loaded nanocarriers were effectively internalized into human lung carcinoma cells, exhibiting synergistic therapeutic effects.

In another interesting study, c(RGDyK)-conjugated SLNs targeted to tumor angiogenic vessels by encapsulating IR-780 iodide dye—a photothermal agent—to monitor PTT by NIR imaging were designed [98]. The conjugation of IR-780 SLNs with CRG was demonstrated through a slight variation in the hydrodynamic diameter (126 nm vs. 145 nm) and zeta potential (−13 mV vs. −3 mV) before and after surface modification, respectively. These factors are typically used as indicators of successful particle modification. The multifunctional cRGDIR-780 SLNs showed desirable monodispersion, significant stability under physiological conditions, and specific accumulation in glioblastoma tumor tissue, demonstrating potential applicability in the targeted and NIR-guided treatment of PTT.

In recent years, the use of magnetic SLNs (MSLNs) has also garnered significant interest as a targeted drug delivery system, demonstrating effective application in cancer treatment. Indeed, by incorporating magnetic nanoparticles (MNs), MSLNs can be guided to tumor sites via magnetic orientation within the bloodstream. Consequently, this approach reduces toxicity and dosage requirements while enhancing reliability, validity, and patient compliance [99]. A recent study introduced a reproducible methodology for preparing spherical MSLNs containing Fe_3_O_4_ cores incorporated in a glyceryl trimyristate solid matrix. These versatile nanotools exhibit promising features for hyperthermia treatment against colon cancer [100]. Notably, in vitro experiments confirmed their hemocompatibility, magnetic responsiveness, and hyperthermia capacity.

The authors of another study [101] proposed Technetium-99m (_99_mTc), with a short half-life and suitable gamma energy emission, to label solid magnetic lipid nanoparticles loaded with azathioprine (AZA). Radiolabeling is a crucial technique used in nuclear medicine for diagnosis and treatment, which allows the tracking of drug behavior in vivo by replacing the atoms of a drug compound with a radioisotope. The characterization of the radiolabeled SLNs revealed a mean diameter of 205 nm and a zeta potential of −14 mV, suggesting that the nanoparticles exhibited minimal propensity for aggregation.

Based on the literature reviewed, the proposed formulations of SLNs exhibit biocompatibility, biodegradability, and safety. These characteristics create new perspectives in the landscape of nanomedicine and hold significant promise for future treatments and monitoring. SLNs enable the targeted delivery of therapeutic agents and in vivo imaging. However, further in vivo studies are necessary to fully assess and validate the potential of SLNs as theranostic tools.

### 3.3. Nanostructured Lipid Carriers (NLCs)

Nanostructured lipid carriers (NLCs), with a general diameter range from 10 to 1000 nm, are a second-generation smart transport system developed to overcome the limitations of SLNs, including limited drug loading capacity and drug expulsion during storage [97,102]. NLCs consist of a binary mixture of solid and liquid lipids that, like SLNs, have low toxicological potential and are solid at both room and body temperature. They can be produced by several techniques that require high energy input, such as high-pressure homogenization (HPH), high shear homogenization/sonication, supercritical fluids, and microwave-based techniques. NLCs contain partially crystallized lipid droplets and a less-ordered crystalline structure or an amorphous solid structure, ensuring high encapsulation efficiency and physical stability [103]. They also represent a valuable option to improve the chemical stability, bioavailability, and controlled release of functional lipophilic compounds entrapped in the lipophilic core [104,105].

Traditionally, NLCs have been used as carriers for sustained-release drugs. However, recent studies highlighted their potential in medical imaging. A recent work by Li and colleagues [22] involved a dual-function NLC designed for breast cancer treatment. Named IR780-AMD-NLCs, this system combines two essential features: an outer shell coated with AMD3100 (a chemokine receptor antagonist) for precise tumor targeting and an inner core that encapsulates the hydrophobic IR780. The IR780-AMD-NLCs demonstrated high IR780-loading capacity (approximately 95%) and efficient AMD3100 coating (around 73%). Importantly, the integration of the IR780 into the NLCs enhanced its properties, resulting in improved heat generation efficiency compared with free IR780 under repeated laser irradiation. Moreover, the surface coating of the NLCs with the AMD3100 allowed for high accumulation in tumors and showed an interesting photothermal anti-tumor effect and anti-metastatic efficacy in vivo.

In a recent investigation [106], NLC was radiolabeled with Technetium-99 m tricarbonyl complex (_99_mTc (CO)^3^), with an encapsulation efficiency and drug loading equal to 49% and 0.5%, respectively. The carrier exhibited a mean diameter of approximately 240 nm and a zeta potential of around −34 mV. Importantly, the _99_mTc (CO)^3^-PTX-loaded NLC was interesting for detecting overexpressed folate receptors in various tissues using gamma imaging after intravenous injection.

In another study [23], NLCs were successfully designed and formulated for the targeted delivery of PTX and ICG to combine chemotherapy with photodynamic therapy. The hydrodynamic diameters of blank NLCs and drug-loaded NLCs were less than 100 nm, with a PDI value lower than 0.2. This drug delivery system enhanced drug stability and produced sufficient local reactive oxygen species (ROS) for the effect of NIR laser stimulus, and increased intracellular drug uptake, resulting in enhanced cytotoxicity in tumor cells.

More recently, Olerile et al. [25] developed a novel co-loaded nanostructured lipid carrier loaded with QDs and PTX for parenteral administration, with the goal of providing necessary data for preclinical studies on the formulation. The particle size was found to be approximately 115 nm, whereas the encapsulation efficiency was lower than 90%.

A theranostic approach for continuous treatment response monitoring is advantageous in assessing chemotherapy effectiveness. In this context, functionalizing nanocarriers with magnetic nanoparticles, such as iron oxide nanoparticles (IONP), facilitates tumor and metastasis visualization in various organs [106]. For instance, in a recent study [107], researchers developed NLCs loaded with DTX-MNLC (docetaxel and iron oxide nanoparticles) for lung cancer treatment. The DTX-MNLC formulation showed a particle size ranging from 10 to 200 nm, with an entrapment efficiency exceeding 99%. However, to demonstrate the effectiveness and safety of the formulation, further in vivo tests are required.

Until now, research studies have demonstrated that while theranostic NLCs show great promise, especially in terms of continuous monitoring and targeted treatment, their full potential remains to be explored. In vivo studies are essential for validating their safety, efficacy, and practical application. Therefore, in the near future, researchers will need to bridge the gap between in vitro findings and real-world scenarios to ensure a successful clinical translation.

### 3.4. Lipid Nanocapsules (LNCs)

Lipid nanocapsules (LNCs) are camouflaged nanocarriers with a design that combines features of both liposomes and polymeric nanoparticles. LNCs are generally composed of an oily core of medium-chain triglycerides, enclosed in an amphiphilic outer layer comprising a PEGylated surfactant and, alternatively, lecithin or other co-surfactants. LNCs are characterized by sizes between 20 and 100 nm [108].

Basu et al. [109] produced LNCs co-encapsulating PTX and salinomycin (SAL) for breast cancer cells and cancer stem cells, respectively. The LNCs were produced by the phase inversion temperature (PIT) method, and they showed a mean particle diameter of 90 nm and negative zeta potential (−7 ± 3 mV). LNCs loaded with the active principle exhibited a monomodal particle size distribution, with a PDI smaller than 0.3, and 98% drug encapsulation efficiency.

The aim of the study by Gupta and coworkers [110] was to develop solid-lipid-core nanocapsules (SLCNs) comprising a polymeric corona coated with PEG for the co-delivery of PTX and erlotinib (ERL) to non-small cell lung cancer, and to examine their physicochemical properties and in vitro behavior. The SLCNs showed an average diameter of 196 ± 3 nm, a PDI equal to 0.215, and a zeta potential of −29.8 ± 0.4 mV. The formulation led to the clearly defined early and late apoptosis of cancer cells, exhibiting more marked effects than those resulting from the individual free drugs.

Gupta et al. [111] also developed folate-receptor-targeted hybrid lipid-core nanocapsules comprising a hybrid lipid core loading tanespimycin (TNP) and a polymeric corona of DOX (F-DTN). These lipidic systems could deliver DOX and TNP sequentially to study their in vitro release. The F-DTN showed remarkable morphological properties, with highly monodispersed particles. The average particle diameter of the F-DTN was 208 ± 3 nm, with a PDI of 0.211, and an average zeta potential equal to −16.4 ± 1.8 mV. The encapsulation efficiency of the F-DTN for the TNP was equal to 98.15 ± 0.19% *w*/*w*, whereas that for the DOX was equal to 96.07 ± 0.73% *w*/*w*. The in vitro tests proved excellent cytotoxicity, high cellular uptake, and apoptosis.

In another study, Lollo et al. [112] developed lipid nanocapsules (LNCs) for curcumin (CCM) encapsulation by the phase inversion method. They produced LNCs loaded with CCM with a mean diameter of 50 nm and a zeta potential of −8 ± 3 mV. The CCM exhibited hydrophobic properties and a high affinity for the hydrophobic cores of LNCs, achieving an encapsulation efficiency of approximately 90%. The in vitro tests demonstrated that the CCM-loaded LNCs induced significant apoptotic effects on the glioma cells, enhanced internalization, and stimulated release.

Szwed et al. [113] produced DiD (1,1′-dioctadecyl-3,3,3′,3′ tetramethylindocarbocyanine 4-chlorobenzenesul fonate)-labeled LNCs and studied the mechanisms of action that achieve the intoxication of breast cancer cells. The average particle sizes of the LNCs, both with and without DiD, ranged from 92 to 95 nm, with a PDI of less than 0.05 and a zeta potential in the range of −7 to −10 mV. The encapsulation efficiency of the DiD was 100%. These findings demonstrated that the action mechanism of the LNCs was highly dependent on the cell type.

The aim of the study developed by Balzeau et al. [114] was to prepare LNC and LNC–paclitaxel using an emulsion inversion phase technique. The mean diameter of the particles was 56 ± 0.1 nm for the LNC and 55 ± 0.1 nm for the LNC–paclitaxel. The PDI for the different formulations was always lower than 0.2, suggesting a singular population of spherical nanoparticles. The zeta potential was slightly negative (−8.7 ± 1.2 mV and −8.5 ± 1.9 mV for the LNC and LNC–paclitaxel, respectively), owing to the presence of PEG on the surface. Furthermore, the study investigated the capacity of NFL-TBS.40-63 peptide to target mouse glioma cells when it was matched with lipid nanocapsules; the NFL-TBS.40-63 peptide exhibited the ability to improve the delivery of lipid nanocapsules in mouse glioma cells, resulting in an innovative approach based on its ability to penetrate into cells.

### 3.5. Lipid Nanoemulsions (NEs)

Nanoemulsions are special kinds of nanoparticle, typically ranging in size between 50 and 500 nm, which have attracted interest in recent years because of their efficient ability to load a large variety of therapeutic and imaging agents [115]. The term “nanoemulsion” is used to describe the dispersions of water and oil, two immiscible liquids that create a thermodynamically stable and isotopically transparent system. Nanoemulsions can be easily prepared using high shear pressure, or by the mechanical extrusion system [98]. To disperse droplets in an aqueous phase, emulsifying agents, including surfactants/co-surfactant and additives, are employed to stabilize the system [116]. These are compounds with an amphiphilic profile that reduce the interfacial tension between two immiscible phases, as they are constituted by hydrophobic bicarbonate tails, which tend to place themselves in non-polar liquids, and a polar head, which usually places itself in the polar liquid [117]. The ingredients of a nanoemulsion are approved as safe (GRAS) by the FDA, making the formulation highly safe to administer, with enhanced bioavailability and therapeutic efficacy [118].

Their nanoscale size, extensive surface area, high drug loading capacity, and favorable drug release profile make nanoemulsions highly relevant for cancer treatment [116,119]. Recently, several strategies involving the use of nanoemulsions have been suggested to selectively affect the tumor microenvironment for both diagnostic and therapeutic purposes [120].

In a recent study, Patel et al. [121] developed a novel system against ovarian cancers. Over 80% of ovarian cancers have supraexpressed folate receptor-α (FR-α). Therefore, folate (FA) was considered to be an active targeting moiety for FR-α^+^ ovarian cancer. To improve therapeutic efficacy and minimize toxicity, docetaxel (DTX)-loaded FA-NE labeled with gadolinium (Gd) was developed, because Gd allows real-time pharmacodynamic data to be acquired on the accumulation of the anticancer system in target lesions. In that study, the NEs showed a size distribution of about 150 nm and a zeta potential, influenced by the presence of mPEG 2000-DSPE in the formulations, of around −45 mV. The active targeting of FA was evaluated against ovarian cancer cells, resulting in a significant enhancement in cell association, which was surface-ligand-density-dependent.

In a more recent study [122], lipid nanoemulsions (LNs) decorated with folic acid (PTX/DHA-FA-LNs) were produced for the co-delivery of PTX and docosahexaenoic acid (DHA), with the aim of fighting breast cancer. Notably, the PTX/DHA-FA-LNs, along with the PTX/DHA-LNs, exhibited a particle size of less than 200 nm and a PDI below 0.3. This size range allows for the passive targeting of tumor tissues through the enhanced permeability and retention effect. Moreover, the encapsulation efficiency of the PTX and DHA was impressive, with the PTX achieving 96% and the DHA 92%. In vitro release experiments demonstrated controlled drug release, whereas cell analyses revealed synergistic effects against breast cancer cells.

In another interesting study [123], a porphyrin–lipid nanoemulsion (PLNE-PTX) prepared by the self-assembly of porphyrin–lipid around a glyceryl trioctanoate oil core was proposed, encapsulating PTX. This innovative platform combines photodynamic therapy facilitated by porphyrin and chemotherapy enabled by paclitaxel. PLNE-PTX nanoemulsions of approximately 120 nm were obtained. The inclusion of a PEGylated lipid to stabilize the nanoemulsion resulted in a biocompatible nanoparticle with a neutral surface (−2.4 mV), high loading capacity for PTX (3.1% *w*/*w*) and porphyrin–lipid photosensitizer (18.3% *w*/*w*), prolonged blood circulation, and excellent accumulation in the tumor. Additionally, the inherent fluorescence of PLNE-PTX allowed the real-time tracking of nanoparticles within the tumor, providing valuable information for treatment planning.

Perfluorocarbon (PFC) nanoemulsions are regarded as attractive systems owing to their capacity to encapsulate therapeutic agents and vaporize into microbubbles and nanobubbles, resulting in the death of cancer cells [124]. Among these, perfluorohexane nanoemulsions (PFH-NEs) stand out as promising ultrasound contrast agents for verifying nanoemulsion payload delivery.

Fernandes and Kolios [27] synthesized PFH-Nes, and their size was influenced by the concentrations of PFH and Zonyl FSP, a fluorosurfactant used to form a stable shell around nanoparticles. By optimizing these concentrations, monodispersed nanoparticles with a mean diameter of approximately 60 nm were produced, which were capable of effectively traversing endothelial gaps within the tumor vasculature. Importantly, the anionic character of the fluorosurfactant rendered the PFH-NEs negatively charged and highly stable (with a zeta potential of about −72 mV), preventing phase separation, flocculation, and coalescence. The optimized PFH-NEs were designed to carry therapeutic agents, such as doxorubicin and paclitaxel. Additionally, nonlinear ultrasound signals from the cells were enhanced by laser-induced PFH bubbles, aiding in treatment tracking. Notably, these PFH-NEs were efficiently internalized in the cancer cells and induced significant cell death.

According to these studies, the use of nanoemulsions as versatile carriers for cancer therapy and imaging appears to be highly encouraging. Nevertheless, nanoemulsions face several critical issues, like their tendency to coalescence and the formation of larger particles, which could limit their theranostic potential. Therefore, it will be essential to face challenges related to their stability. Researchers are actively exploring ways to minimize coalescence and particle growth, with the goal of developing stable theranostic nanoemulsions.

### 3.6. Lipid Micelles

Micelles are composed of surfactants, whose molecules can spontaneously form spherical particles in water. Therapeutic/diagnostic particles can be efficiently encapsulated into the hydrophobic core of micelles, and targeting agents can be loaded onto the hydrophilic surfaces [3]. Moreover, the hydrophilic shell is usually decorated with PEG and can give high solubility and stability to the system [125].

Kang et al. [126] developed a nanocarrier system for theranostic use composed of micelle nanoparticles co-encapsulating PTX and fluorescence QDs (reported as immuno-PTX-QDMs and aptamo-PTX-QDMs). An antibody or an aptamer against the epidermal growth factor receptor (EGFR) was conjugated to the micelle surface to achieve tumor-targeting capacity. The mean size of the PTX-QDMs was equal to 40 ± 3 nm, and the zeta potential was −2.52 ± 0.54 mV. The encapsulation efficiencies of the QDs and PTX were higher than 90%. The efficient accumulation of QDs delivered by the immuno-QDMs and aptamo-QDMs led to the effective optical imaging of target tumors.

Rajendrakumar et al. [127] successfully synthesized a zwitterionic polymeric lipid micelle (PCB–lipid micelle) in which IR-780 dye was encapsulated for real-time particle tracking and PTT. The PCB-lipid-IR-780 nanoparticles exhibited diameters equal to 318 nm, with PDI values smaller than 0.1 and a zeta potential of −0.2 mV. These NPs exhibited exceptional photostability, biocompatibility, and high heat conversion following NIR laser irradiation.

For the precise diagnosis and successful treatment of tumors, Choi et al. [128] developed lipid micellar structures loaded with QDs for cancer theranostics. QD/lipid micelles (QDMs) were produced by a simple self-assembly process and then were conjugated with EGFR antibodies for cancer targeting. Moreover, the micellar nanoparticles were optimized for siRNA delivery (iQDM/siBcl2). The QDMs and iQDMs exhibited spherical shapes, with diameters equal to 50 nm. The diameters of the QDMs, QDM/siBcl2, and iQDM/siBcl2 were 43 ± 5, 41 ± 1, and 32 ± 2 nm, respectively. The zeta potential values of the QDMs, QDM/siBcl2, and iQDM/siBcl2 were 0.63 ± 1.30, −2.85 ± 0.05, and 1.56 ± 0.18, respectively.

In the study by Lu et al. [129], cationic self-assembled N-[1-(2,3-dioleoyloxy) propyl]-N, N, N-trimethylammonium methyl sulfate (DOTAP) and monomethoxy poly(ethylene glycol)-poly(ε-caprolactone) (MPEG-PCL) hybrid micelles (DMP) were produced to transport Bcl-xl siRNA and Mcl1 siRNA for colon cancer treatment. These DMP micelles were stable and were capable of unifying and transmitting Bcl-xl siRNA and Mcl1 siRNA to colon cancer cells. The particle size distribution showed that the DMP micelles were nanosized, with a PDI equal to 0.31 and an average diameter of 145 nm. The zeta potential was 46.4 mV.

Hu et al. [130] produced a self-assembled folate (FA)-decorated MPEG-PCL micelle to encapsulate curcumin (Cur) (FA/Nano-Cur) for colorectal cancer treatment. The FA/Nano-Cur micelles exhibited a particle size of approximately 30 nm, with a PDI of 0.17 and a zeta potential of −3.55 mV. The FA/Nano-Cur showed a notably better therapeutic effect than the free Cur and Nano-Cur. The treatment with the FA/Nano-Cur resulted in the apoptosis of tumor cells and a significant reduction in the tumor proliferation and cancer angiogenesis.

Howell et al. [131] developed multifunctional lipid-micellar nanoparticles (LMNs) containing manganese oxide (M-LMNs) for MRI and DNA/drug delivery. A polyethyleneglycol–phosphatidylethanolamine (PEG-2000-PE) lipid was loaded into these lipid micelles to provide biocompatibility and permit long blood circulation times. These cationic lipid micelles can encapsulate manganese oxide (MnO) nanoparticles and the theranostic agent, DOX, in the core and DNA on the surface to achieve both MRI contrast and DNA/drug delivery to mark cells. The mean diameter of the M-LMNs was equal to 100 nm. These micelles exhibited a net positive zeta potential of 37 mV.

In summary, micelles are potential nanoparticles for theranostics thanks to their specificity and stability, but their drug loading is limited by their small dimensions.

In Table 2, the main features and the trial statuses of the different case studies analyzed in this review are summarized.

## 4. Clinical Barriers in Lipid-Based Nanotheranostics

Despite the significant research on lipid-based nanoparticles as drug delivery systems and therapeutic vehicles, no lipid-based nanotheranostic formulation has yet received FDA approval. In fact, although lipids are classified as GRAS, resulting in biocompatibility and expendability to mitigate the toxic effects of some chemotherapeutic drugs, challenges persist in the clinical translation of lipid-based nanotheranostics [132]. Lipid nanotheranostics against cancer face challenges due to personalized chemotherapy, specific size criteria (≤100 nm), and considerations such as protein binding, biodistribution, and toxicity [133]. Currently, only a few liposomal formulations are being explored in the clinical stage of progress [134]. In this regard, as reported by Naziris et al. [133], only one liposomal formulation is under clinical evaluation for chemotherapy in breast cancer, specifically in phase II. Three other liposomal formulations are in phase I. Among the various challenges faced by lipid nanotheranostics for cancer, the large-scale production of complex nanoplatforms is difficult due to variations in physical and chemical characteristics, low yields, and reproducibility in the final properties. In addition, nanotheranostics combine diagnostic and therapeutic components whose optimal delivery may vary. Hence, achieving synergistic effects requires a careful balance of both aspects. Another major concern is ensuring the safety profile of nanotheranostics in humans, which requires the long-term monitoring of patients during clinical trials. The lack of clear FDA policies specific for nanotheranostics also poses a non-negligible regulatory challenge [9].

Clinical applications of nanomedicine require an intensive and thorough characterization of the associated properties, since minor changes in the chemistry or manufacturing process may result in major changes in biodistribution and tolerability in vivo. However, it is possible to avoid clinical translation failures by establishing strict criteria (such as testing and quality control) when designing and developing nanomedicines [135].

### Challenges and Strategies in the Development and Clinical Translation of Lipid-Based Nanocarriers

In the following section, the main challenges that the development of lipid-based nanocarriers face in their translation to clinical practice are discussed. Additionally, potential strategies are proposed to overcome these obstacles and accelerate the advancement of lipid-based nanotheranostics from the bench to the bedside.

(1) Physicochemical stability and scalability. Ensuring the stability of lipid-based nanocarriers during storage and handling is a critical task. Indeed, lipid-based nanocarriers frequently encounter stability concerns, such as aggregation, fusion, and leakage of encapsulated drugs. These issues can be addressed by optimizing the lipid composition, adjusting the types and ratios of lipids used, and incorporating appropriate stabilizing agents, enhancing the structural stability and integrity of nanocarriers [134,136]. Furthermore, scaling up the production of lipid-based nanocarriers while maintaining quality and consistency is a significant challenge. Some approaches to overcoming these difficulties include developing reproducible processes to ensure consistency during large-scale production [136] and implementing strict quality control measures at each step of the manufacturing process to ensure that the final product meets the required standards [134]. These strategies have the potential to significantly enhance the stability and scalability of lipid-based nanocarriers, making them more feasible for widespread application in theranostics.
(2) High costs. The high costs associated with lipid-based nanocarriers represent a great challenge for clinical translation. Some of the primary factors that contribute to these expenses are associated with manufacturing procedures, scale-up expenses arising from the intricate nature of specialized equipment and processes required for the transition from laboratory to full-scale production, and the cost of raw materials [133,137]. There are several strategies that could be used to overcome these problems. For instance, the implementation of more efficient production techniques can help to distribute expenses and streamline development procedures. In addition, it could be useful to obtain funding from government grants and private investors to provide the necessary financial support for research.
(3) Long-term monitoring of patients. Lipid nanocarriers pose several challenges in clinical translation. For instance, comprehending the distribution of these nanocarriers within the body, their safety profile, and their clearance is of utmost significance [134]. The adoption of personalized approaches based on individual patient characteristics and the use of advanced imaging techniques to monitor the distribution of nanocarriers within the body could be turning points for the widespread and safe application of lipid nanocarriers. Moreover, the utilization of lipid nanocarriers may pose a risk of immune reactions, which may complicate their long-term usage [138]. Therefore, it is essential to conduct extensive preclinical testing to assess the safety of immunological agents.
(4) Complex regulatory pathways. The approval process for nanomedicines is extremely complex. The lack of specific guidelines for nanocarriers can lead to significant delays. However, advanced in vitro and in vivo models can provide more reliable data on the safety and efficacy of nanomedicines, helping to overcome regulatory hurdles. It is essential to develop regulatory guidelines specifically for nanomedicines in order to streamline the approval process [134]. Therefore, establishing appropriate guidelines and regulations will be essential for a safe and effective clinical translation. Collaboration is essential to advance lipid-based nanocarriers in cancer therapy. By combining resources, data, and expertise, academia, industry, and regulatory agencies can tackle common challenges more effectively. This collaborative approach can significantly improve the clinical translation process, ensuring that promising therapies reach patients faster. Joint research initiatives have the potential to yield novel solutions, as well as a deeper understanding of the intricate processes involved in the creation and approval of advanced theranostic products.

## 5. Conclusions

Lipid nanocarriers have emerged as versatile and powerful tools in the field of theranostics, offering a unique combination of diagnostic and therapeutic capabilities. Their biocompatibility, ability to encapsulate a wide range of therapeutic and diagnostic agents, and potential for surface modification make them ideal candidates for targeted and personalized medicine. Continued research in the development and synthesis of chemically modified LNPs to achieve tunable biodegradability in vivo would allow the optimization of delivery vehicles, making them more versatile, highly efficient, and biocompatible. Advances in nanoparticle design have significantly improved their stability, targeting efficiency, and controlled-release properties, addressing some of the major challenges in current medical treatments. It is important to note that, among the various types of lipid nanoparticles, liposomes are currently the only lipid-based carriers for theranostics that are undergoing clinical trials. This highlights their established safety profile and efficacy, setting a milestone for other lipid nanoparticles in the theranostic field.

However, despite the promising results, several hurdles remain to be overcome before lipid nanoparticles can be widely adopted in clinical practice. These include their potential toxicity, the scalability of their production, regulatory approval, and the need for more comprehensive in vivo studies to fully understand their long-term effects and efficacy. Furthermore, combining diagnostic and therapeutic functionalities into a single nanoparticle system poses additional challenges in terms of design and functionality.

For these reasons, the use of LNPs in medicine is expected to expand significantly. The development of different types of LNPs with optimized drug delivery properties, such as nanostructured lipid carriers and ionizable cationic nanoparticles, brings additional advantages to LNP formulations and broadens the perspectives of their applications [139].

Future research should focus on the development of more sophisticated nanoparticle systems, improving targeting accuracy, and ensuring safe and cost-effective production methods. Interdisciplinary collaboration among chemists, biologists, engineers, and medical professionals will be crucial to advance this technology from the laboratory to the clinic.

In conclusion, lipid nanoparticles hold great potential for revolutionizing the field of theranostics by pioneering more efficient and individualized treatment approaches. The continual exploration and development of these nanomaterials could significantly improve the elucidation and management of numerous health conditions, enhancing patient outcomes and standard of living.

The expansion of LNP technologies in other fields is also evident. For example, some cosmetic products are already on the market, and many more are under development. Researchers in further areas, such as nutrition, nutraceuticals, agrochemistry, and nanoreactors are already exploring the advantages of LNPs. Thanks to the recent developments and successes, LNPs can be recognized as being among the most advantageous and promising forms of nanotechnology.

## Figures and Tables

**Table 1:** Theranostics: diagnostic and therapeutic applications.

Treatment	Application	Features	Therapeutic/Imaging Agents
CT	Diagnostic	3D imaging Deep tissue penetration Clear view of cross-sectional images High-resolution imaging High dose of ionizing radiation	Iodine [8] Gold [9] Platinum [10] Bismuth [11] Tantalum [12] Ytterbium [13]
MRI	Diagnostic	Non-invasive High spatial resolution Evaluation of anatomic details Clear view of the cross-sectional images Good soft-tissue contrast Low sensitivity Expensive	Manganese (Mn^2+^) [14] Iron (Fe^3+^) [15] Gadolinium (Gd^3+^) [16]
PET	Diagnostic	Non-invasive Low spatial resolution High sensitivity 3D imagining Unlimited penetration depth Quantitative analysis Use of radioactive probes (toxic potential)	^11^C [17] ^64^Cu [18] ^68^Ga [19] ^13^N [20] ^18^F [21]
NIR	Diagnostic	Non-invasive Non-ionizing Deep tissue penetration Low tissue absorption and scattering High fluorescence intensity Low sensitivity	IR-780 [22] ICG [23] Quantum dots [24,25] Gold NPs [26]
USI	Diagnostic	Real-time measurement Non-ionizing and non-radio labeling High temporal and spatial resolution Low sensitivity US-frequency-dependent depth penetration	Perfluorocarbon [27] Gold NPs [28] Carbon NPs [29] Polymer NPs [30]
PDT	Therapeutic	Minimally invasive Selective targeting of tumors Photo-responsive Oxidative stress due to photodynamic effect	Tetrapyrrole family [31] ICG [32] Quantum dots [33]
PTT	Therapeutic	Non-invasive Low toxicity Conversion of photo energy to thermal energy High specificity Low size effect	Carbon dots [34] Graphene, iron oxide, carbon nanotubes, gold, silver [6]
Chemotherapy	Therapeutic	Tumor reduction Toxic therapeutic agents Side effects	Doxorubicin [35] Paclitaxel [36]
Magneto-Thermal Therapy	Therapeutic	Minimally invasive Deep tumor targeting Safe Side effects	Iron oxide [37]
Ultrasound Responsive Therapy	Therapeutic	Non-invasive Efficient drug delivery Side effects	Perfluorocarbon [38]
Radiotherapy	Therapeutic	Ionizing radiation Precise targeting Radiotoxicity	Gold nanorods [39]

**Table 2:** Lipid nanocarriers for theranostics.

Nanocarriers	Theranostic Agent	Production Technique	Size [nm]	ζ-Potential [mV]	Trial Status	Ref.
Liposomes	DOX + ICG	Lipid film hydration method	130	−39	In vivo	[84]
	ICG	Thin film/extrusion method	80	-	In vivo	[85]
	CA4P + IONP	Reverse phase evaporation method	209	-	In vivo	[86]
	Gd	Lipid film hydration method	113	−58	In vitro	[87]
	PCX + VNB	Lipid film hydration method	190	−9	In vivo	[88]
	IMT	Lipid film hydration method	250	54	In vitro	[89]
SLNs	PTX + siRNA + QDs	Emulsification solvent evaporation method	130	36	In vitro	[24]
	IR-780 dye	Slightly modified solvent diffusion method	145	−3	In vivo	[98]
	Fe_3_O_4_	Double emulsion/solvent evaporation method	180	−40/20	In vitro	[100]
	AZA + ^99^mTc + FeO/Fe_3_O_4_	Solvent diffusion method	205	−14	In vitro	[101]
NLCs	IR-780 dye	Solvent evaporation method	156	−48/−12	In vivo	[22]
	PTX + ^99^mTc(CO)_3_	Solvent diffusion method	237	−34	In vitro	[106]
	PTX + ICG	Solvent diffusion method	100	-	In vivo	[23]
	PTX + QDs	Oil/water emulsification solvent evaporation technique	115	-	In vivo	[25]
	IONP + DTX	Solvent injection technique	110	-	In vitro	[107]
Lipid Nanocapsules	PTX + SAL	Phase inversion temperature method	90	−7	In vitro	[109]
	PTX + ERL	Nanoprecipitation/sonication	196	−30	In vivo	[110]
	DOX + TNP	Mixing/sonication techniques	208	−16	In vitro	[111]
	CCM	Phase inversion technique	50	−8	In vitro	[112]
	DiD	Solvent free phase inversion method	95	−10	In vivo	[113]
	PTX	Emulsion inversion phase process	55	−9	In vitro	[114]
NEs	DTX + Gd	High-shear homogenization method	150	−45	In vivo	[121]
	PTX + DHA	Microfluidic technique	187	-	In vivo	[122]
	PTX + porphyrin	Sonication method	120	−2	In vivo	[123]
	PFH	Sonication method	61	−72	In vitro	[27]
Micelles	PTX + QDs	Thin-film method	41	−3	In vivo	[126]
	IR-780 dye	Sonication method	318	−0.2	In vivo	[127]
	QDs + siRNA	Lipid film hydration method	32	+2	In vivo	[128]
	siRNA	Lipid film hydration method	145	+46	In vivo	[129]
	Cur	Lipid film hydration method	31	−4	In vivo	[130]
	MnO	Lipid film hydration method	100	+37	In vivo	[131]
